# Growth of Biological Complexity from Prokaryotes to Hominids Reflected in the Human Genome

**DOI:** 10.3390/ijms222111640

**Published:** 2021-10-28

**Authors:** Alexander E. Vinogradov, Olga V. Anatskaya

**Affiliations:** Institute of Cytology, Russian Academy of Sciences, 194064 Saint Petersburg, Russia; anatskaya@incras.ru

**Keywords:** evolution of complexity, gene phylostratigraphy, transcription factors, epigenetic factors, nervous system, cancer, diseases, aging

## Abstract

The growth of complexity in evolution is a most intriguing phenomenon. Using gene phylostratigraphy, we showed this growth (as reflected in regulatory mechanisms) in the human genome, tracing the path from prokaryotes to hominids. Generally, the different regulatory gene families expanded at different times, yet only up to the Euteleostomi (bony vertebrates). The only exception was the expansion of transcription factors (TF) in placentals; however, we argue that this was not related to increase in general complexity. Surprisingly, although TF originated in the Prokaryota while chromatin appeared only in the Eukaryota, the expansion of epigenetic factors predated the expansion of TF. Signaling receptors, tumor suppressors, oncogenes, and aging- and disease-associated genes (indicating vulnerabilities in terms of complex organization and strongly enrichment in regulatory genes) also expanded only up to the Euteleostomi. The complexity-related gene properties (protein size, number of alternative splicing mRNA, length of untranslated mRNA, number of biological processes per gene, number of disordered regions in a protein, and density of TF–TF interactions) rose in multicellular organisms and declined after the Euteleostomi, and possibly earlier. At the same time, the speed of protein sequence evolution sharply increased in the genes that originated after the Euteleostomi. Thus, several lines of evidence indicate that molecular mechanisms of complexity growth were changing with time, and in the phyletic lineage leading to humans, the most salient shift occurred after the basic vertebrate body plan was fixed with bony skeleton. The obtained results can be useful for evolutionary medicine.

## 1. Introduction

The growth of complexity in evolution is a most intriguing phenomenon that has value in terms of the formation of worldviews, specifically in relation to the meaning of life, because the growth of complexity continues in human society. This is a long-debated problem [1]. The concept of ‘orthogenesis’ (Scala Naturae, Natural Ladder, the chain of beings, progressive evolution, etc.) has become obsolete, yet an increase in complexity in certain lineages, as well as simplification in some others, can be recognized. There is no immanent vector towards an ultimate complexity goal because natural selection is not teleological and acts only for immediate adaptation.

It is likely that complexity can grow only when all accessible lower ecological niches are occupied (an analogy with atomic orbitals). The ‘atomic orbitals’ model has no relation to orthogenesis. Moreover, it is even the inverse because it suggests a general trend towards simplification due to the high vulnerability, energy and time costs of complex organisms. For instance, the time taken to lose vision (cave animals) is much faster than to acquire it [2]. Mutations are chaotic and more likely to destroy than construct organization. Hence, mutation pressure should act towards simplification (except for gene and genome duplication). The more complex organisms are under weaker purifying selection, which counteracts the pressure of deleterious mutations, implying that their genes are less optimized because of a higher burden of slightly deleterious mutations [3]. In more complex organisms, purifying selection is biased in favor of the ‘information technology’ of life (the regulation of gene expression and development) but, overall, it is still weaker [3]. Therefore, it seems plausible that complexity can grow only when accessible lower niches are occupied. Conversely, when lower niches become vacant, the simplification can proceed, which is also observed in evolution [4,5].

Although it is notoriously difficult to define complexity [6,7,8], many attempts have been made. Genome size and the number of genes were earlier assumed as possible molecular measures, yet anatomically simpler organisms may contain larger genomes and higher numbers of genes than more complex creatures; these phenomena are called C-value and G-value paradoxes [9]. Additional DNA in the genome probably has other functions besides informational and regulatory (e.g., buffering of chromatin structures from environmental fluctuation), which can explain the large variation of its amount in anamniotes, invertebrates, and plants [10]. Later, the features of noncoding DNA, including the length of untranslated mRNA, gene expression, cell differentiation, alternative splicing, protein structural disorder, number of cell types, encephalization quotient, and anatomical brain and heart complexity, and the properties of purifying selection, were proposed [3,8,11,12,13,14,15,16,17]. Although there is still no gold standard for measuring complexity, the above-listed indicators (particularly the number of cell types, encephalization quotient, and brain and heart complexity) show that multicellular organisms are generally more complex than unicellular organisms, amniotes than anamniotes, and mammals than reptiles.

The road to complexity was probably not smooth and might have been governed by different factors at different evolutionary stages. For instance, it was supposed that at small phylogenetic distances (i.e., last evolutionary stages), alteration in gene expression can be more important than changes in protein-coding sequences [18,19]. The gene repertoire (the proportion of certain gene groups in the genome) and complexity-related gene properties can be the other factors.

Besides fundamental aspects, this problem is important for medicine because cancer is considered as an evolutionary reversal (atavistic shift) to unicellularity [20,21]. The genes of unicellular origin are overexpressed in cancer tissues, whereas the genes appearing at multicellular evolutionary stages are downregulated [22,23,24]. The human interactome (protein interaction network) contains two giant clusters that are strongly enriched in the genes either of unicellular or multicellular origin and their corresponding functions, which indicates the existence of a multicellular/unicellular contrast in cellular networks [25]. The genes downregulated with human age are enriched in the unicellular cluster, whereas the upregulated genes are overrepresented in the multicellular cluster.

The clusters have denser interactions within than between them; therefore, they can serve as attractors (stable states of dynamic systems) of cellular programs. Importantly, the unicellular cluster has a higher inside/outside connection ratio compared with the multicellular cluster, which suggests a stronger attractor effect and may explain why cells of multicellular organisms are prone to oncogenesis [25]. The unicellular cluster is activated in human cancers, which was shown in the single-cell transcriptomes of various cancer types with the control for cell cycle activity [26]. These data suggest that oncogenesis is not just an alteration in a few genes but the switching to ancient unicellular programs (when cells tend to behave as independent organisms).

From the viewpoint of the evolution of complexity, a reversal to unicellularity is a downshifting along the complexity axis, which can be considered as a manifestation of a general vector to simplification due to mutation pressure. This consideration makes the study of the growth of complexity in evolution important, as it can become a part of the evolutionary medicine framework [27,28]. The understanding of the molecular mechanisms of this growth may help to elucidate the etiology of diseases and aging, and even suggest possible remedies. Thus, certain unicellular-specific drugs can be applied for the treatment of cancer [29,30]. Similarly, the evolutionary history of the genetic basis of other diseases and aging can improve our understanding of their causes and suggest possible cures. This approach can also be important for regenerative medicine. The regenerative capacity is higher in simpler organisms [31,32]. Therefore, the controlled activation of earlier metazoan programs may facilitate injury healing and rejuvenation.

This work is an attempt to obtain a picture of molecular mechanisms that promoted the growth of complexity in the long run from prokaryotes to hominids. We used phylostratigraphy (first proposed in [33]) of human genes to reveal changes, which can be associated with an increase in complexity. Our conclusions are related only to the phyletic lineage leading to humans. Because of the goals of evolutionary medicine, our emphasis was not on the evolution of complexity per se, but specifically on its traces in the human genome.

## 2. Results

### 2.1. Gene Repertoire Approach

As a proxy to biological complexity, which can be reflected in the human genome, we considered the intricacy of regulation. It is well-known that complex organization requires complex regulation [34,35]. With the increase in the number of system elements, the number of possible system states grows exponentially [36]. Thus, the regulatory problems grow faster than the number of regulated elements. Therefore, we chose several of the most inclusive gene groups related to various cellular and organismal regulatory systems for analysis. They are as follows: transcription and epigenetic factors (regulation at the level of transcription, i.e., at the first stage of gene expression), protein modifiers and kinases (regulation at the gene product level, i.e., at the last stage of gene expression), signaling receptors (regulation at the level of intercellular communication), and the nervous system-related genes (regulation at the whole-organism level).

For possible relevance to evolutionary medicine, we studied also several gene signatures of cancer, tumor suppression, pluripotency, aging, and diseases. Tumor suppression, aging, and diseases are important problems of multicellular organization, and are probably exacerbated with the growth of complexity and the related increase in body size and longevity (because diseases indicate vulnerabilities in a complex organization).

**Figure 1:**
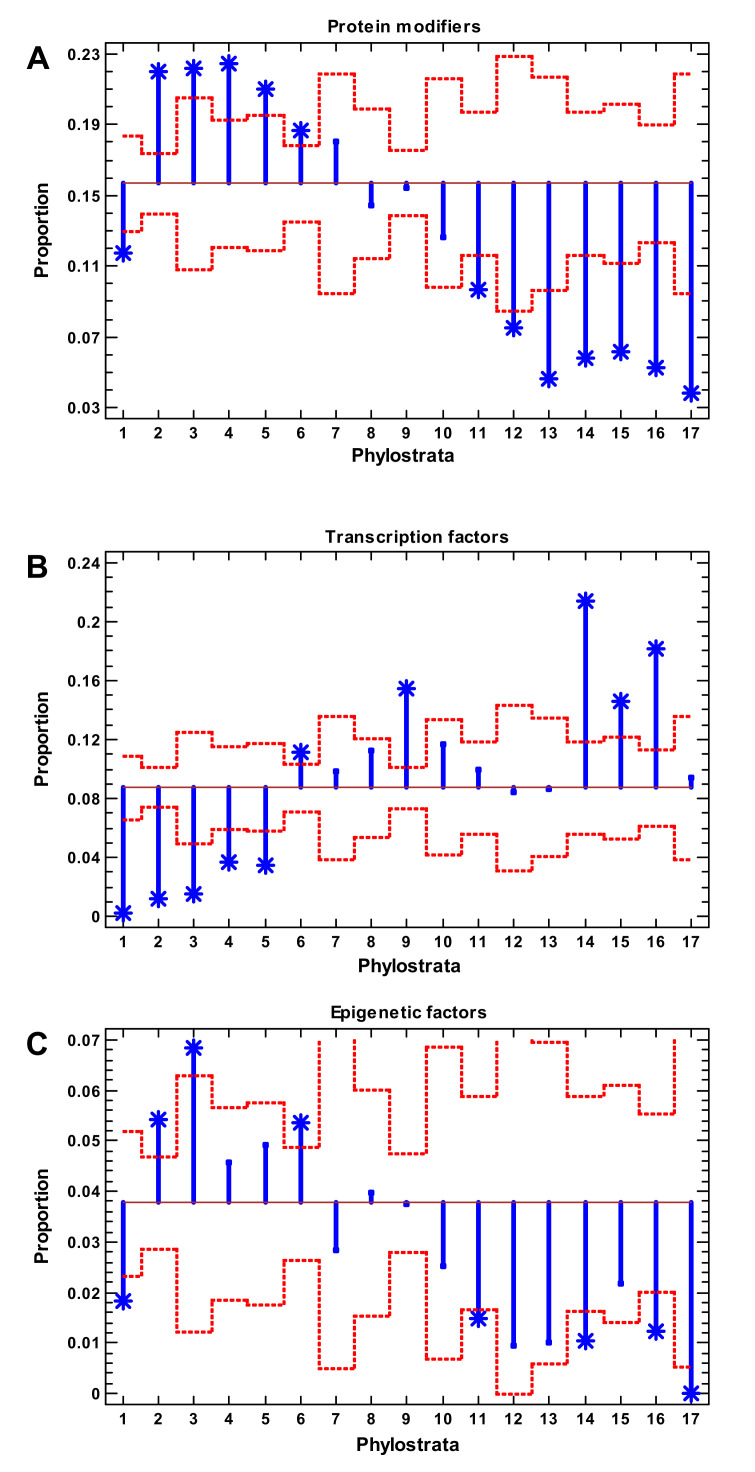
Gene proportion analysis of protein modifiers (**A**), transcription factors (**B**), and epigenetic factors (**C**). Red dotted lines show significance level (*p* < 0.05), asterisks show significant enrichment (if above baseline) or underrepresentation (below baseline). (1—cellular organisms; 2—Eukaryota; 3—Opisthokonta; 4—Metazoa; 5—Eumetazoa; 6—Bilateria; 7—Chordata; 8—Vertebrata; 9—Euteleostomi; 10—Tetrapoda; 11—Amniota; 12—Mammalia; 13—Theria; 14—Eutheria; 15—Boreoeutheria; 16—Primates; 17—Hominidae.)

**Figure 2:**
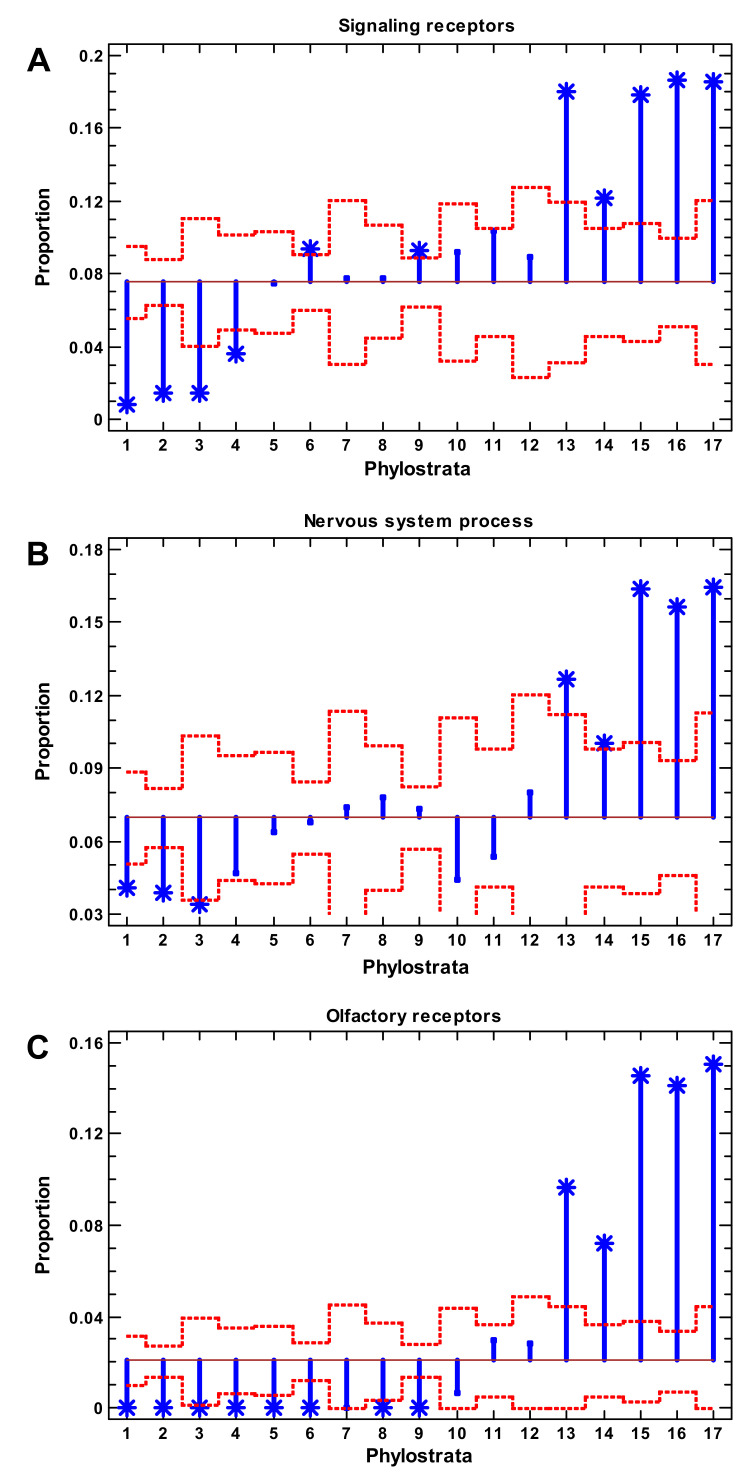
Gene proportion analysis of signaling receptors (**A**), nervous system process genes (**B**), and olfactory receptors (**C**). Red dotted lines show significance level (*p* < 0.05), asterisks show significant enrichment (if above baseline) or underrepresentation (below baseline). (1—cellular organisms; 2—Eukaryota; 3—Opisthokonta; 4—Metazoa; 5—Eumetazoa; 6—Bilateria; 7—Chordata; 8—Vertebrata; 9—Euteleostomi; 10—Tetrapoda; 11—Amniota; 12—Mammalia; 13—Theria; 14—Eutheria; 15—Boreoeutheria; 16—Primates; 17—Hominidae.)

To reveal the rise and fall of different gene groups in evolution, we analyzed the proportion of genes belonging to a tested gene group in the sets of genes that originated at different phylostrata (evolutionary stages). This proportion was compared with the proportion of genes belonging to this group in the whole genome. The whole-genome proportion was considered as a baseline for a given gene group. The statistical significance of enrichment depends both on the change in proportion and the number of genes in a phylostratum. Therefore, the line indicating the significance level can vary across phylostrata even when there is a similar enrichment in gene proportion (Figure 1 and Figure 2).

### 2.2. Protein Modifiers and Transcription Factors

Somewhat counterintuitively, the protein modifiers showed the earliest growth of regulatory complexity, albeit they affected the latest stage of gene regulation (at the level of gene products). Their wave began in the unicellular eukaryotes, showing a plateau at the Eukaryota-Metazoa (recent unicellulars and sponges) and a depression after the Chordata (Figure 1A) A similar picture was observed with the protein kinases (a subset of protein modifiers), albeit their depression began somewhat earlier (Supplementary Figure S1A).

The transcription factors (TF), which operated at the earliest stage of gene regulation, gave rise to the second wave of complexity (Figure 1B). They were underrepresented up to Eumetazoa (recent cnidarians) and showed significant enrichment at the Bilateria (vertebrates and main invertebrate lineages) with their maximum at the Euteleostomi (bony vertebrates). Then, a recession began. However, there was a second expansion of TF at the Eutheria, which will be analyzed in detail later. A similar picture was observed with the two other TF databases (Supplementary Figure S1B,C). Despite the fact that TF had already appeared in the Prokaryota (which do not have chromatin), the epigenetic factors expanded earlier than TF, with a depression after the Bilateria (Figure 1C). A similar picture was observed with the TF cofactors, whose expansion also predated the rise of TF (Supplementary Figure S2A).

As there is growing knowledge that chaperones (assisting in protein folding) play a regulatory role [37], we analyzed their phylostratic course. They showed an early expansion with a sharp decline after the Metazoa, i.e., slightly predating the expansion of protein modifiers, with an earlier decline (Supplementary Figure S2B). The bivalent genes, which have both activating and repressive epigenetic marks in their promoters and, therefore, can switch on/off quickly, are also believed to be important regulators [38]. Their growth generally coincides with the first expansion of TF (Supplementary Figure S2C).

### 2.3. Signaling Receptors, Nervous System, and Olfactory Receptors

The signaling receptors represented intercellular communications. They showed the wave in general coinciding with the expansion of TF (Figure 2A). The nervous system-related genes formed the latest expansion, with the enrichment beginning from Theria (marsupials and placentals) (Figure 2B). It was intuitively appealing (and with support from the encephalization quotient [15]) that the nervous system should play an increasing role in the growth of complexity. Therefore, it was tempting to assume that the expansion of nervous system-related genes presented the last wave of complexity. However, the enrichment of both signaling receptors and nervous system-related genes in the later phylostrata was due to olfactory receptors (participating in both gene groups), which showed a sharp expansion beginning from Theria (Figure 2C).

**Figure 3:**
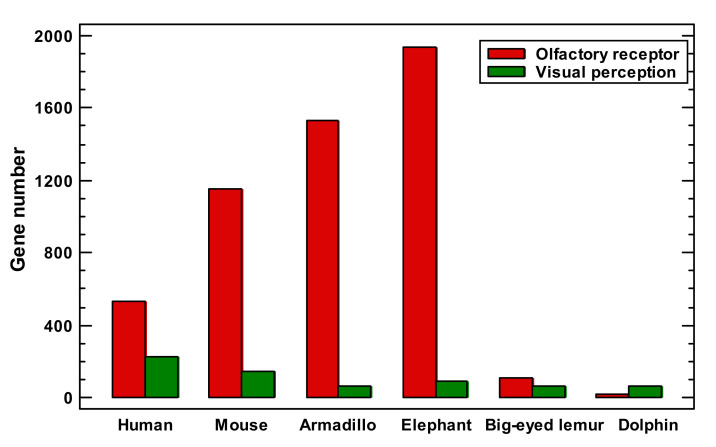
The numbers of olfactory receptors and visual-perception genes in different mammals.

**Figure 4:**
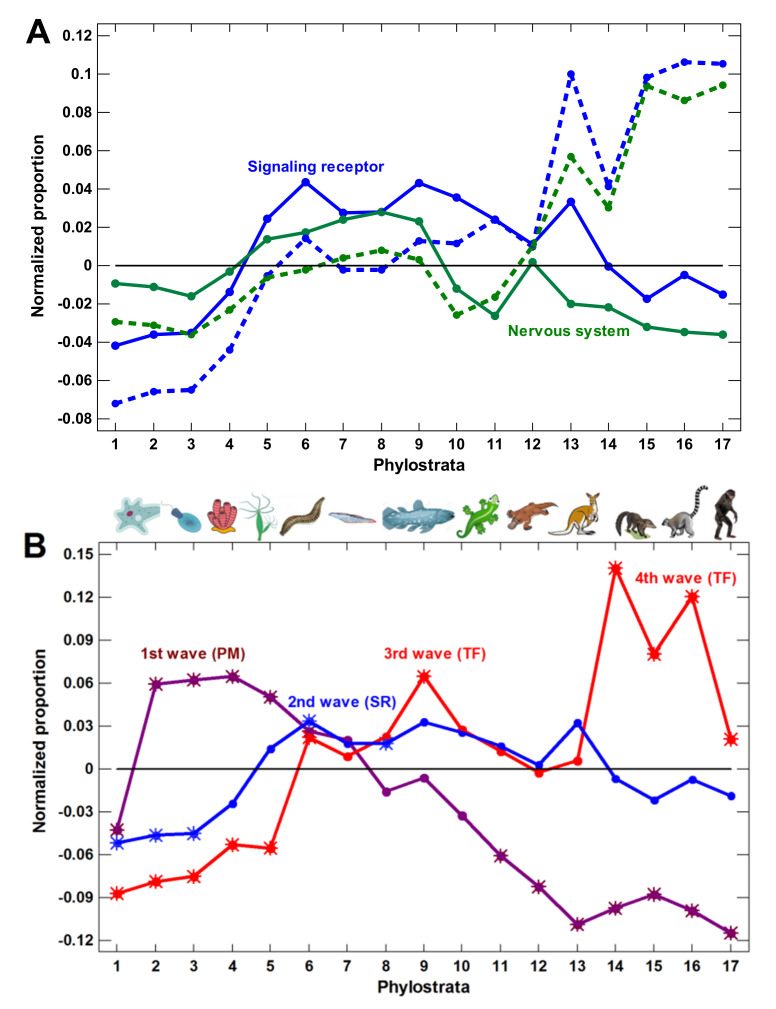

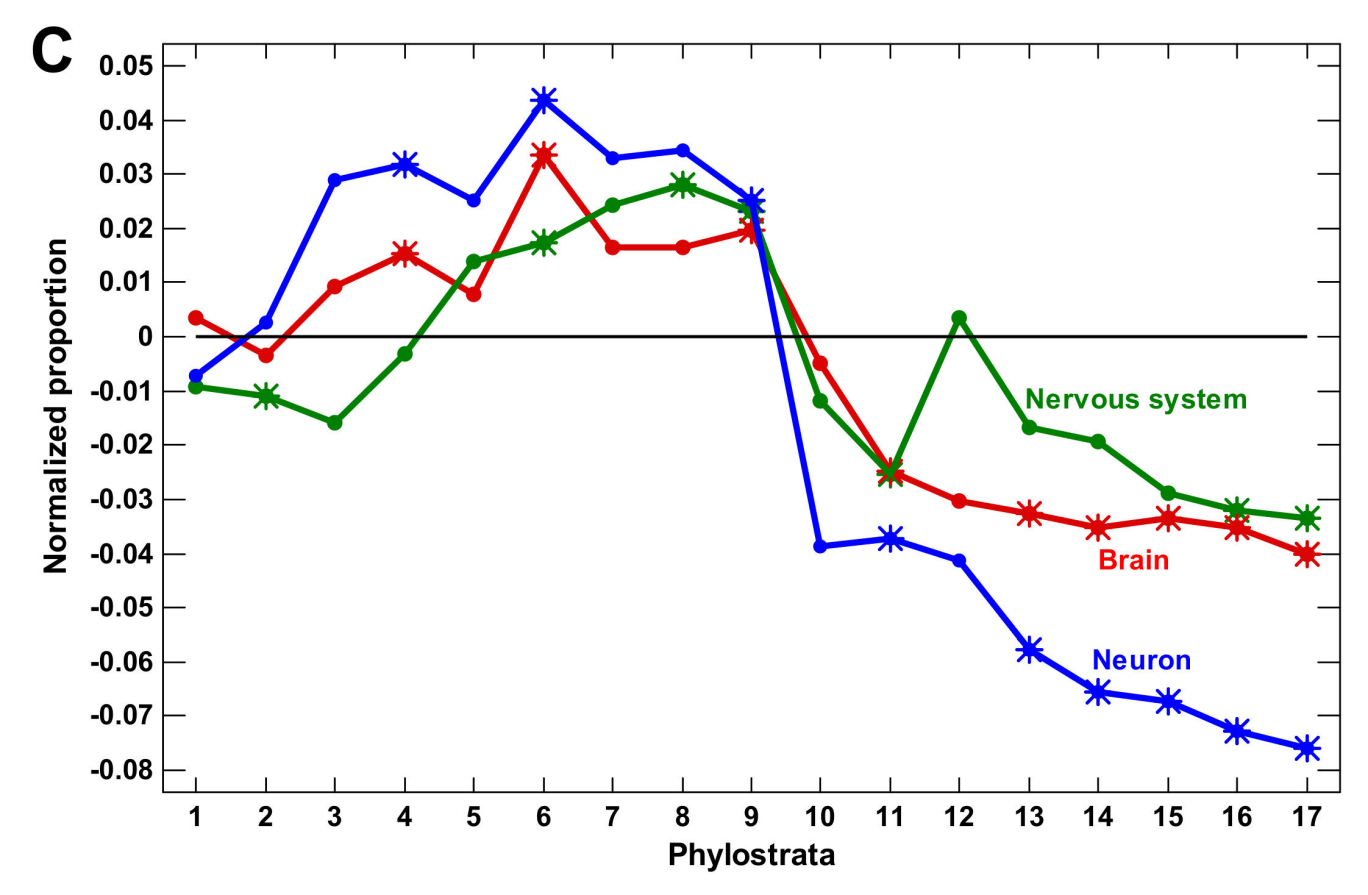
Phylostratic course of normalized gene proportions for different gene groups (baselines for all groups are set to zero). (**A**) signaling receptors and nervous system process genes (dotted lines, w/o exclusion of olfactory receptors; solid lines, with exclusion). (**B**) Waves of regulatory complexity: protein modifiers (PM), signaling receptors (SR), and transcription factors (TF). (**C**) Different sets of nervous system-related genes. Asterisks show significant enrichment (if above baseline) or underrepresentation (below baseline). (1—cellular organisms; 2—Eukaryota; 3—Opisthokonta; 4—Metazoa; 5—Eumetazoa; 6—Bilateria; 7—Chordata; 8—Vertebrata; 9—Euteleostomi; 10—Tetrapoda; 11—Amniota; 12—Mammalia; 13—Theria; 14—Eutheria; 15—Boreoeutheria; 16—Primates; 17—Hominidae.)

This point needs special consideration. The olfactory system is unique in that each odor requires a special receptor and each olfactory sensory cell expresses a specific receptor [39]. Therefore, the complexity and intricacy of the olfactory system depend upon a disproportionately large number of genes compared with the visual system [39]. The number of olfactory receptors in different mammals varies above 100-fold, whereas the number of genes belonging to the visual system differs only up to threefold (Figure 3). Taking into account the high variation in the number of olfactory receptors, which is not relevant to general complexity (additional arguments are given in the Section 3), we analyzed the impact of olfactory receptors on the enrichment of signaling receptors and nervous system genes. Without olfactory receptors, both gene sets showed a decline in the latest phylostrata instead of an expansion (Figure 4A).

### 2.4. General Picture

For an accurate picture, as we excluded the olfactory receptors from the signaling receptors and the nervous system genes, we also needed to exclude them from the total gene set because their exclusion from a particular gene group without exclusion from the genomic background may have distorted the picture. The results obtained with the total exclusion of olfactory receptors are shown in Figure 4B. The consolidated picture includes the four waves of regulatory complexity (Figure 4B). The signaling receptors began to expand somewhat earlier than TF; therefore, we marked them as the second wave (after the first wave of protein modifiers). The phylostratic course of the nervous system genes without olfactory receptors is supported by two other nervous system-related gene sets (GO categories containing the words ‘brain’ or ‘neuron’) (Figure 4C). (Here, there was no need to exclude olfactory receptors because they are not mapped to these GO categories.) All three nervous system-related gene sets declined after the Euteleostomi (Figure 4C). The nervous system genes showed a minor (not significant) elevation at the Theria, which mostly involved the taste and pheromone receptors (Supplementary Table S1).

Generally, up to Euteleostomi, the expansion of all three nervous system-related gene sets coincided with the growth of other regulatory genes. This was probably because the nervous system should integrate all the other complexity waves at the organismal level (hence, we did not mark it as a separate wave). As additional tests, we analyzed the sets of genes belonging to ‘central nervous system development’, ‘multicellular development regulation’, and any GO process containing a word ‘regulation’. All these gene sets declined after the Euteleostomi (Supplementary Figure S3).

### 2.5. Tumor Suppressors, Oncogenes, Pluripotency, Aging, and Diseases

**Figure 5:**
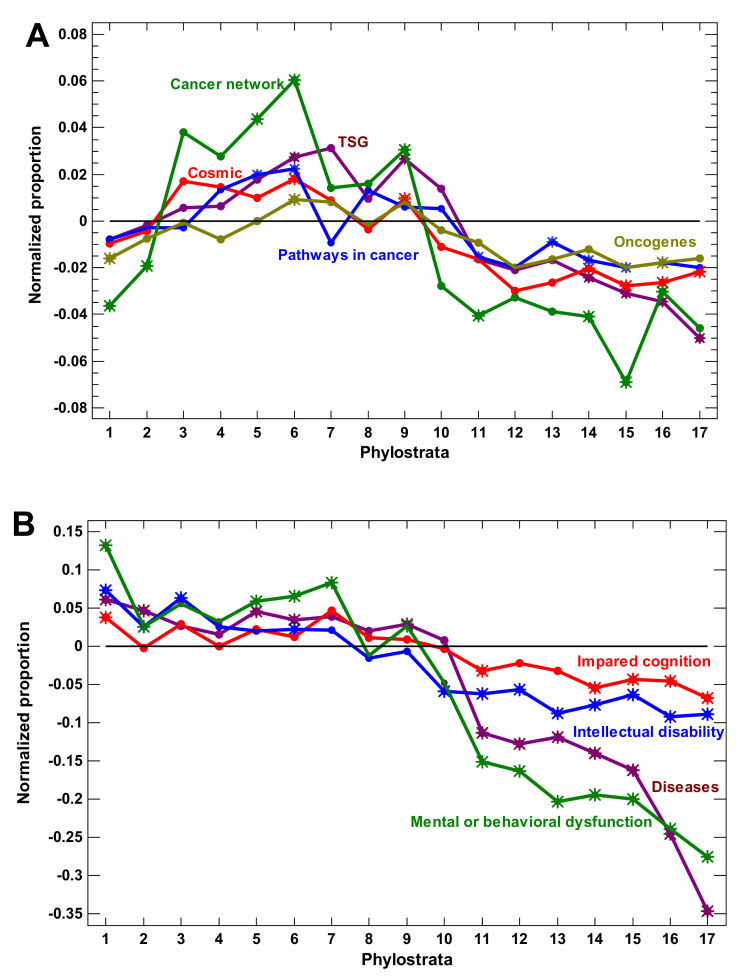
Phylostratic course of normalized gene proportions for different gene groups (baselines for all groups are set to zero). (**A**) Cancer-related genes. (**B**) Disease-related genes. Asterisks show significant enrichment (if above baseline) or underrepresentation (below baseline). (1—cellular organisms; 2—Eukaryota; 3—Opisthokonta; 4—Metazoa; 5—Eumetazoa; 6—Bilateria; 7—Chordata; 8—Vertebrata; 9—Euteleostomi; 10—Tetrapoda; 11—Amniota; 12—Mammalia; 13—Theria; 14—Eutheria; 15—Boreoeutheria; 16—Primates; 17—Hominidae.)

Tumorigenesis is a most important problem in multicellular organization, which reflects the main contradiction between the cellular and organismal levels [25]. Therefore, it directly pertains to organismal complexity. The various cancer-related signatures (including tumor suppressors) expanded in the multicellular phylostrata with a decline after the Euteleostomi (Figure 5A). The most comprehensive signature (‘cancer network’) showed its maximum at the Bilateria.

The pluripotency signatures (see Section 4) reflecting the developmental potential showed the opposite pattern, with their maximum in the unicellular organisms and their decline in the multicellular organisms (Supplementary Figure S4), which is reminiscent of the biogenetic (recapitulation) principle. Similarly, the genes downregulated with age showed their maximum in the unicellular organisms and their decline in the multicellular organisms. The age-upregulated genes showed no significant maximum (Supplementary Figure S4).

The disease-related genes can be important for complexity because diseases indicate vulnerabilities in a complex organization. Notably, they are highly enriched in regulatory genes (e.g., for enrichment in ‘regulation of multicellular organismal process’, GO:0051239; *p* < 10^−219^; hypergeometric test). The disease-related genes declined after the Euteleostomi (Figure 5B). With regard to regulatory complexity, neural disorders are of special importance. The various gene sets altered in neural disorders also decline after the Euteleostomi (Figure 5B), corroborating the previous analysis of nervous system-related genes (Figure 4C).

### 2.6. Second TF Expansion and Deep Phylostratigraphy

The second expansion of TF looked appealing with regard to the growth of complexity (as the nervous system genes looked before the exclusion of olfactory receptors). A sharp increase in TF abundance was observed at the Eutheria (Figure 4B). They were mostly C2H2 zinc fingers, many containing the KRAB domain (Supplementary Table S2). It would be tempting to suggest their relation to complexity. However, birds have far less ZF-KRAB genes than reptiles, and the crocodile, which is the closest reptile to birds, shows an intermediate number (Supplementary Table S3). Turtles are the oldest extant reptiles, and they have the largest population of ZF-KRAB genes (20-fold higher than birds). These observations indicate that abundance of ZF-KRAB genes is related to clades rather than to grades of complexity.

**Figure 6:**
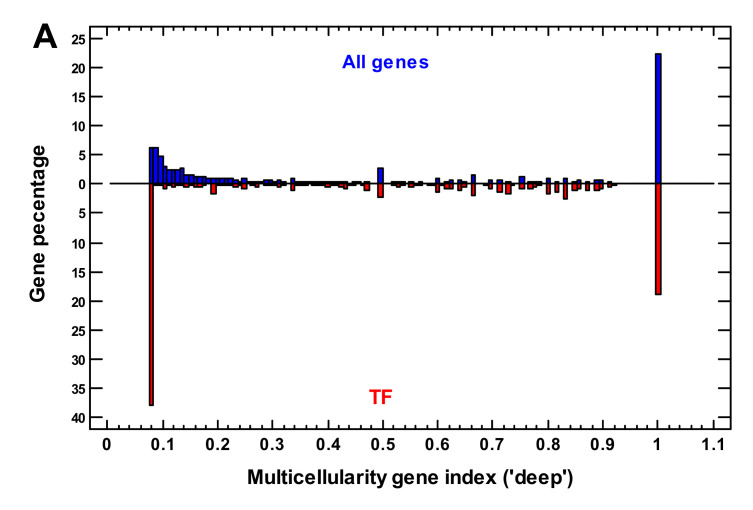

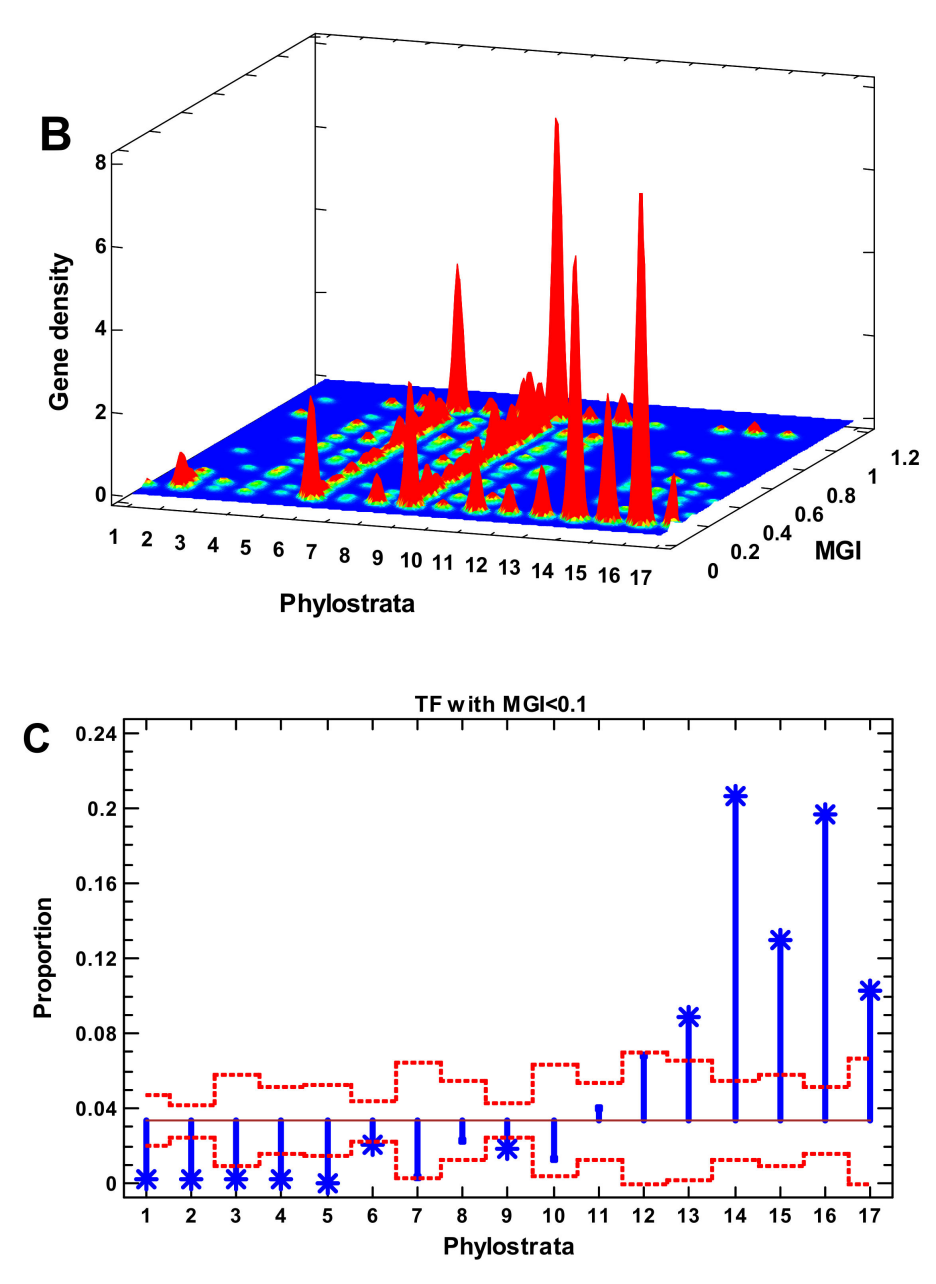
Analysis of transcription factors (TF) using deep vs. shallow phylostratigraphy. (**A**) Gene density distribution of multicellularity gene index (MGI) for all genes and for TF. (**B**) Bivariate gene density distribution of deep vs. shallow parameters for TF, (**C**) gene proportion analysis for TF with MGI < 0.1. (1—cellular organisms; 2—Eukaryota; 3—Opisthokonta; 4—Metazoa; 5—Eumetazoa; 6—Bilateria; 7—Chordata; 8—Vertebrata; 9—Euteleostomi; 10—Tetrapoda; 11—Amniota; 12—Mammalia; 13—Theria; 14—Eutheria; 15—Boreoeutheria; 16—Primates; 17—Hominidae.)

We applied deep phylostratigraphy (see Section 4) to estimate the primary origin of these TF. In contrast to the shallow approach that uses only strict orthologs, the deep phylostratigraphy approach considers the more relaxed orthologous gene groups (OGG), which also include paralogs. It allows the determination of the earliest traceable origins of gene ancestors. We constructed the multicellularity gene index (MGI), which showed the proportion of multicellular organisms in the OGG, to which a human gene belongs (Figure 6A). In other words, it showed how often the relaxed orthologs occurred in multicellular organisms compared with unicellular organisms. If the MGI was equal to 1, the related genes were not found in unicellular organisms, if the MGI was low (<<1), the related genes were observed more often in unicellular organisms than in multicellular organisms. The human TF showed two main groups: the first group was more typical for unicellular organisms (MGI < 0.1), and the second group was of purely multicellular origin (MGI = 1), with minor TF fractions in the phylostrata in between the two main groups (Figure 6A).

The bivariate density distribution shows the expansions of TF from the shallow vs. deep viewpoints (Figure 6B). Although the KRAB domain appeared only in the Tetrapoda [40], the TF of the last expansion had a deeply unicellular origin (MGI < 0.1) (Figure 6B). These TF (with MGI < 0.1) were enriched only in the latest phylostrata (Figure 6C). On the contrary, the TF that are typical of multicellular organisms were enriched in the Bilateria-Euteleostomi (Supplementary Figure S5). Thus, here we see a paradoxical inversion: the TF of the latest expansion were of the earliest origin.

To be more exact, TF with MGI < 0.1, albeit underrepresented, still exist in the Bilateria-Euteleostomi (Figure 6B,C). There are also C2H2 zinc fingers, many containing a BTB/POZ domain or a SET domain (Supplementary Table S4). They also include EGR1-3 genes that are important for brain development.

The difference between the deep and shallow phylostratigraphy can be better explained through the example of ohnologs (genes retained in pairs after whole genome duplication, WGD). All ohnologs are of pre-WGD origin by deep definition. However, after duplication, many genes experience sub- or neofunctionalization [41]. Hence, they become the new genes by shallow definition. Such genes should change their protein sequence to a greater extent than the genes retaining initial functions. Therefore, they cannot be reciprocal best hits to the genes from the previous phylostrata and are mapped to the post-WGD phylostrata by shallow phylostratigraphy. Notably, in this study, TF did so more often than the other genes (Supplementary Figure S6). The two rounds of WGD occurred between Chordata and Vertebrata [42]. Therefore, all ohnologs mapped to post-Chordata should have undergone sub- or neofunctionalization. TF are the most enriched ohnologs in post-Chordata (Supplementary Table S5).

### 2.7. Gene Properties

**Figure 7:**
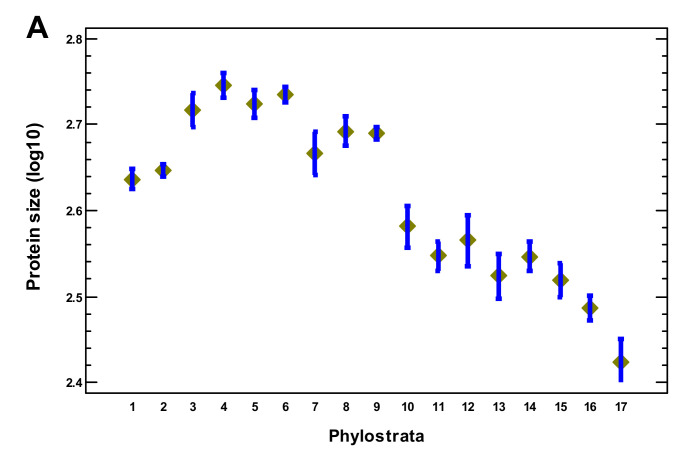

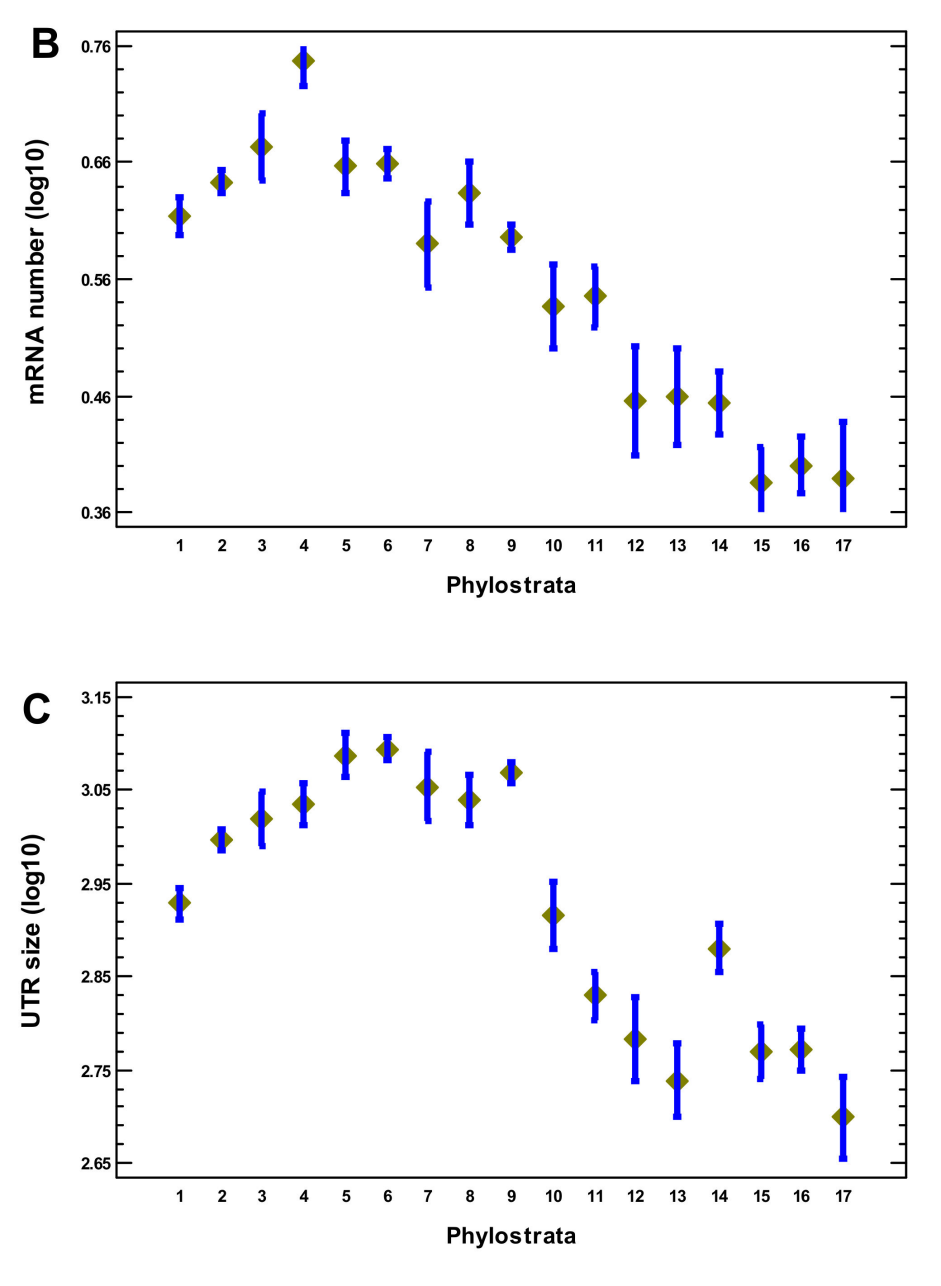
Phylostratic course of complexity-related gene properties. (**A**) Protein size. (**B**) Number of alternative splicing mRNA variants per gene. (**C**) Length of mRNA untranslated regions (UTR). Means with LSD intervals (*p* < 0.05). (1—cellular organisms; 2—Eukaryota; 3—Opisthokonta; 4—Metazoa; 5—Eumetazoa; 6—Bilateria; 7—Chordata; 8—Vertebrata; 9—Euteleostomi; 10—Tetrapoda; 11—Amniota; 12—Mammalia; 13—Theria; 14—Eutheria; 15—Boreoeutheria; 16—Primates; 17—Hominidae.)

**Figure 8:**
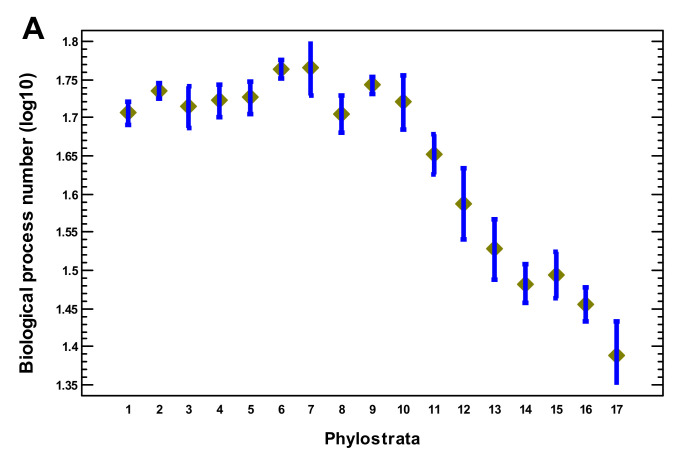

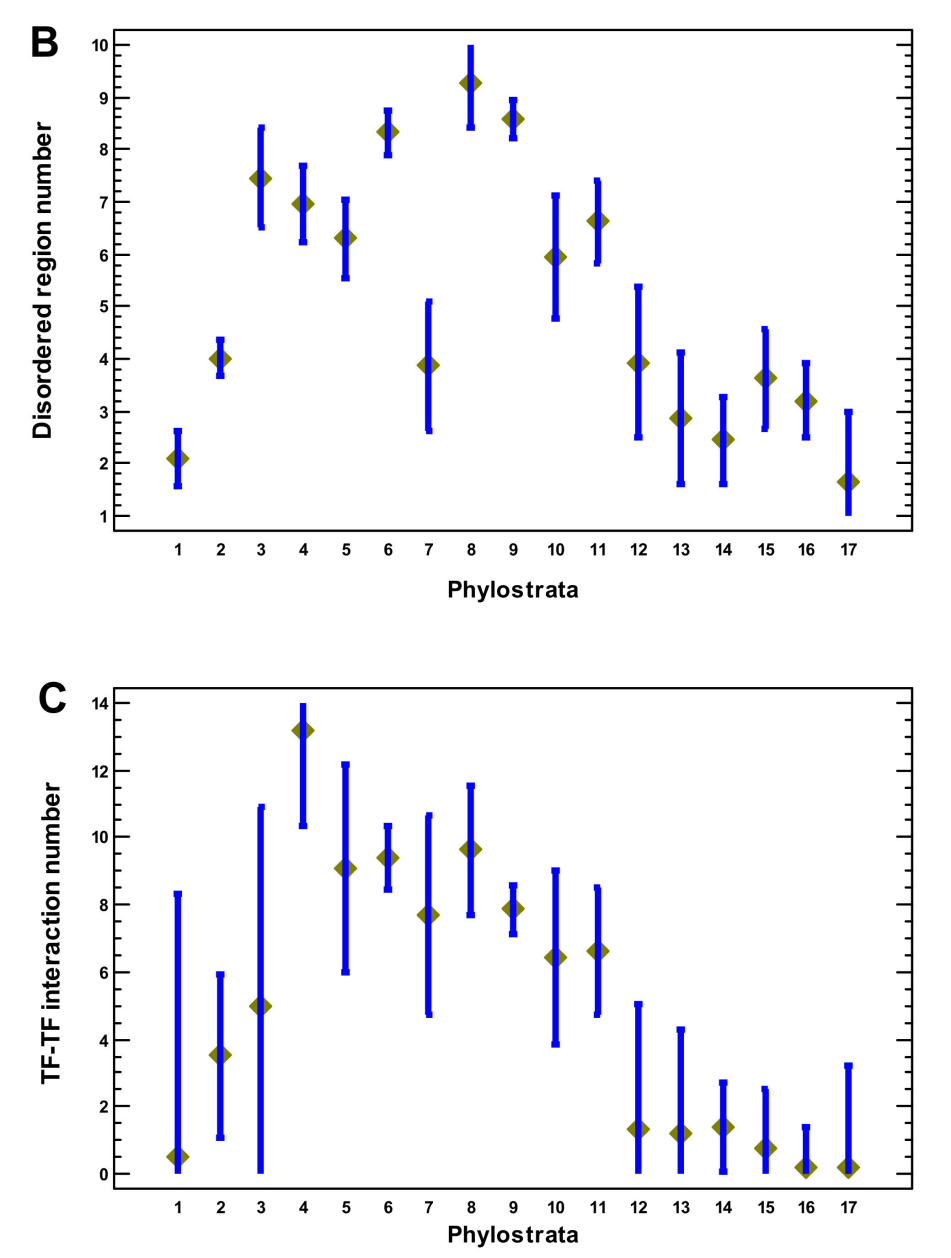
Phylostratic course of complexity-related gene properties. (**A**) Number of GO biological processes in which a gene participates. (**B**) Number of disordered regions in a protein. (**C**) Number of TF–TF interactions. Means with LSD intervals (*p* < 0.05). (1—cellular organisms; 2—Eukaryota; 3—Opisthokonta; 4—Metazoa; 5—Eumetazoa; 6—Bilateria; 7—Chordata; 8—Vertebrata; 9—Euteleostomi; 10—Tetrapoda; 11—Amniota; 12—Mammalia; 13—Theria; 14—Eutheria; 15—Boreoeutheria; 16—Primates; 17—Hominidae.)

In this section, we analyze gene properties in relation to complexity. The lengths of the encoded proteins showed their maximum at the Opisthokonta-Bilateria (Figure 7A). The number of mRNA alternative splicing variants per gene peaked at the Metazoa followed by a gradual decline (Figure 7B). The lengths of untranslated mRNA regions, which can be related to translational regulation [43], showed a plateau with their highest values at the Eumetazoa-Bilateria, and there was a sharp decline after the Euteleostomi (Figure 7C). The number of biological processes per gene plateaued up to the Euteleostomi, with a decline after that (Figure 8A). The number of disordered regions in a protein is thought to relate to complex regulation and was even proposed as a measure of biological complexity [17]. This parameter peaked at the Vertebrata (Figure 8B). As it is known that separate TF may be insufficiently specific for regulation of a particular gene and should work together [44], we checked the density of the TF–TF interactions. This density showed its maximum at the Metazoa and declined later (Figure 8C). The sharpest decline coincided with the second expansion of TF, which suggests that this expansion was not related to the increase in complexity. The number of tissues where a gene was expressed gradually declined from the Eukaryota, with a sharp decline after the Euteleostomi (Supplementary Figure S7).

### 2.8. Protein-Sequence Evolution

**Figure 9:**
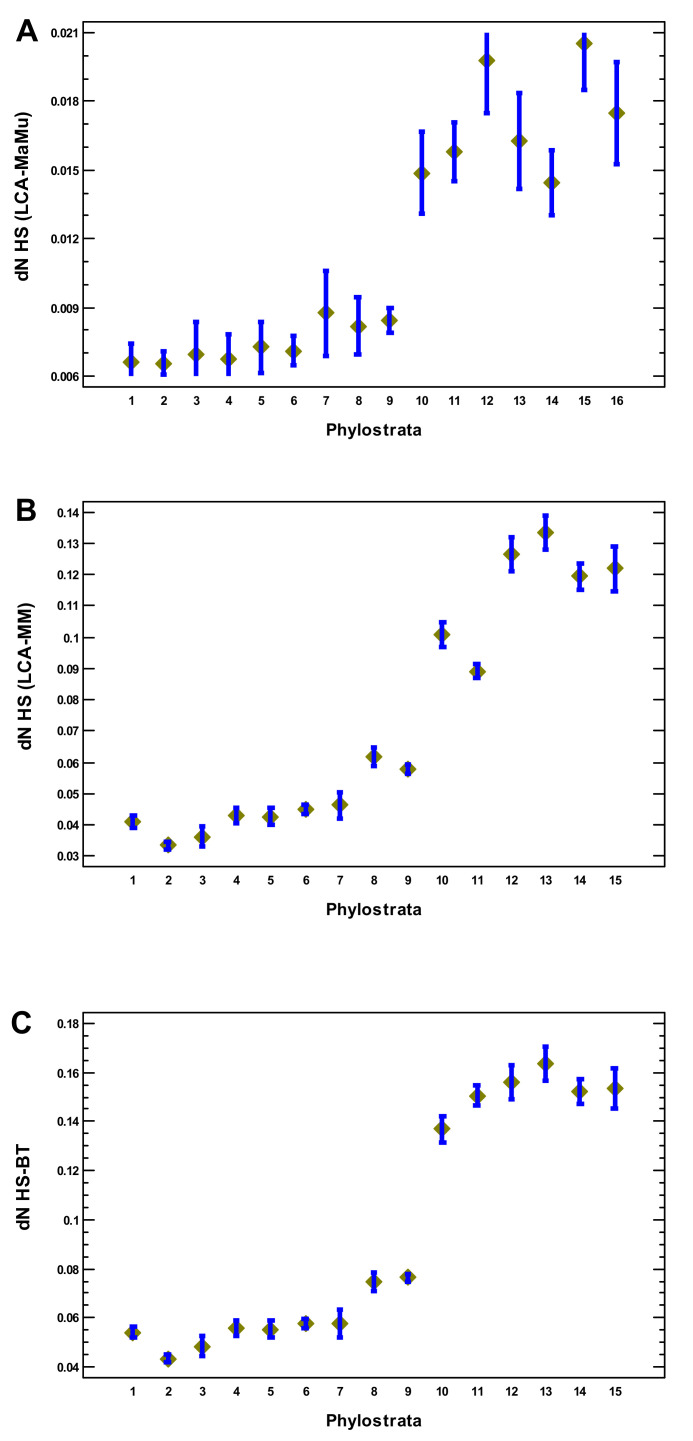
Phylostratic course of protein evolutionary rate (*dN*). (**A**) In the phyletic line from the common ancestor with macaque to the human. (**B**) In the phyletic line from the common ancestor with mouse to the human. (**C**) In the direct human–cow comparison. Means with LSD intervals (*p* < 0.05). (1—cellular organisms; 2—Eukaryota; 3—Opisthokonta; 4—Metazoa; 5—Eumetazoa; 6—Bilateria; 7—Chordata; 8—Vertebrata; 9—Euteleostomi; 10—Tetrapoda; 11—Amniota; 12—Mammalia; 13—Theria; 14—Eutheria; 15—Boreoeutheria; 16—Primates.)

Because the complexity-related genomic features studied here abruptly declined after the Euteleostomi, we considered the problem from another angle and studied the rate of protein-sequence evolution in the genes appearing in the different phylostrata. This rate was analyzed at the latest evolutionary stages (placentals) because of the higher accuracy of such comparisons and the higher number of orthologs. In the human–macaque and human–mouse comparisons (where the lineage leading to human was analyzed), the rate of protein sequence evolution was low in the genes that originated up to Euteleostomi, with about a twofold increase in the genes of the later origin (Figure 9A,B). The same can be seen in the raw human–cow comparison (Supplementary Figure S9C).

## 3. Discussion

This work represents an attempt to trace the evolutionary road to organismal complexity that is reflected in the human genome. Certainly, not all genes that appeared during this long path were retained, but those that served as foundations for further complexity growth were preserved. We showed that this growth was determined by different mechanisms at different evolutionary stages.

Chaperones (that assist in protein folding and are involved in regulation) showed the earliest expansion among all regulation-related genes. Their function is close to protein modifiers; hence, we did not consider them as a separate wave. The expansion of protein modifiers (including epigenetic factors) predated an expansion of transcription factors (TF). This phenomenon is paradoxical because TF firstly appeared in prokaryotes [45], which have no chromatin. This was probably because the majority of the biological regulation in prokaryotes was conducted by protein modifiers, which were adopted in the earlier eukaryotes for epigenetic regulation. For instance, histone modifiers—specifically HATs and HDACs—acetylate and deacetylate thousands of other proteins besides histones [46].

Furthermore, prokaryotic TF are mostly repressive, simply hindering the action of RNA polymerase [45], and chromatin plays a similar role in eukaryotes. Yet, in contrast to prokaryotes, the eukaryotic TF fulfill their function via the recruitment of epigenetic factors [45]; therefore, the earlier expansion of epigenetic factors might facilitate the further expansion of TF. In a practical sense, these results are meaningful for medicine. The chaperones, protein modifiers, and epigenetic factors expanded at the unicellular evolutionary stage, whereas the TF and signaling receptors expanded in the multicellular phylostrata (Figure 1 and Figure 2; Supplementary Figures S1–3). Because cancer is an atavistic shift to unicellularity, our observations suggest that chaperones, protein modifiers, and epigenetic factors can be (at least) as important for oncogenesis as TF and signaling receptors.

### 3.1. Signaling Receptors and Nervous System-Related Genes

The expansion of signaling receptors slightly predated the expansion of TF while the growth of the nervous system-related genes roughly coincided with the first expansion of TF (Figure 4B,C). The genes mapped to the ‘neuron’ and ‘brain’ GO categories showed an even earlier expansion during the Metazoa (recent sponges) when there was no nervous system. This was probably because these genes have other molecular functions (related to the membrane), which were later co-opted for the emerging nervous system. This point was discussed in [47].

It is tempting to put forward the apparent expansions of the nervous system-related genes and signaling receptors as the mechanism of complexity growth in the latest phylostrata (Figure 2B). However, these expansions were due to olfactory receptors, which reflect only the elaboration of olfaction but not general complexity. The variation in the number of olfactory receptors vs. the number of visual system genes in mammals supports this conclusion (Figure 3). The dolphin has 100-fold less olfactory receptors than the elephant (Figure 3). However, it stands at the second place after the human in terms of the encephalization quotient, whereas the elephant has an encephalization quotient that is average for mammals [15]. The deletion of olfactory receptors is pronounced in primates that evolved to switch from an odor-dependent to a visual-dependent integration of information [48], which can hardly be considered as a simplification. After the exclusion of olfactory receptors, the nervous system genes sharply declined after the Euteleostomi (Figure 4C). The genes of the nervous disorders showed a similar decline (Figure 5B).

The two main gene family expansions in placentals (olfactory receptors and ZF-C2H2-KRAB TF) were previously reported [49]. The two growth waves of TF in human evolution were also shown [50]. However, we observed the first TF maximum at the Euteleostomi while the previous authors observed this at the Bilateria. This difference can be explained by a difference in phylostratigraphy. It is likely that in the previous work, the slightly relaxed orthologs (see below) were used, and their phylostratigraphy bore an impact of a deeper gene origin. The shallow vs. deep approaches should be discussed in more detail.

### 3.2. Shallow vs. Deep Phylostratigraphy

The difference between these approaches can be illustrated via the example of the whole genome duplication (WGD). All ohnologs (genes retained in pairs after the occurrence of WGD) are of pre-WGD origin by deep definition, yet sub- and neofunctionalization after the occurrence of WGD caused some of them to become the new genes by shallow definition. The two WGD rounds between the Chordata and the Vertebrata [42] help explain the prevalence of TF, in terms of their amount, at the Euteleostomi relative to the Bilateria. TF were the most enriched group of ohnologs mapped to post-WGD phylostrata by shallow definition (Supplementary Table S5). In other words, they were especially prone to sub- and neofunctionalization. While the other ohnologs showed roughly similar numbers at the Bilateria and the Euteleostomi, the TF-ohnologs strongly prevailed at the Euteleostomi (Supplementary Figure S6).

To our knowledge, the shallow vs. deep approaches were not distinguished previously. For instance, a deep phylostratigraphy appeared when the String, eggNOG, or OrthoDB databases were used because their orthologous gene groups include paralogs. These databases were applied for gene dating in a number of works without it being noted that this is a deep dating mechanism (e.g., [51,52]). In such cases, the genes of unicellular origin constitute above half of human genes. For instance, about 70% of genes were traced to unicellular stage in one of the papers that used deep dating [52], whereas only 39% were mapped to unicellular organisms with shallow dating [22]. In the present paper, these values are 76.5% and 31.5%, respectively. The higher (than previously) deep value can be explained by the fact that we used the database with the broadest species coverage, whereas the lower (than previously) shallow value was probably due to the usage of the stricter orthology (see Section 4). Sometimes a variety of databases, including both the strict and relaxed orthology, were used in the same work with an unsurprising variance in terms of results [53].

Such discrepancies could compromise the very idea of gene phylostratigraphy but this is just a lumping and splitting problem. What is a new gene? Semantically (if we put aside technical issues), this is the classical problem of lumping and splitting, which originated in taxonomy but is now also considered in medicine and other domains that deal with hierarchical classifications [54,55]. Here, we extended this problem to the molecular field. A gene can be of a deep early origin, which is traceable using the orthologous gene groups (OGG) that contain paralogs (lumping), but its function may change with time because of duplication followed by sub/neofunctionalization. Therefore, its strict orthologs can be traced only more recently (splitting). Darwin stated that “it is good to have hair-splitters & lumpers” because splitters remind lumpers that variability should not be ignored, while lumpers remind splitters that variability should not be overinterpreted [55]. We distinguished both approaches and applied them separately, using only strict orthologs for shallow definition (splitting) and OGG that contained paralogs for deep definition (lumping). Both viewpoints are presented as a two-dimensional density distribution (Figure 6B), which revealed a paradox of two expansions of TF. The later expansion consists of the genes with a deep unicellular origin, whereas the earlier expansion involved the purely multicellular genes. This observation is important for understanding the role of the second expansion.

### 3.3. Later TF Expansion

At present, it is generally believed that TF with KRAB domains serve to suppress parasitic transposable elements [40]. In collaboration with the other proteins, they cause heterochromatization of DNA regions that contain (retro)transposons [56]. Thus, they present the mechanism of ‘nuclear immunity’. However, some of them were co-opted for general regulatory functions that were related to development [56]. The TF that were devoted only to nuclear immunity were lost or pseudogenized when their cognate transposons became inactive, whereas those that adopted new functions remained [40,56]. Thus, the older KRAB genes are probably loaded with adopted functions. It is plausible that genomic parasites are a permanent problem of cellular life, and therefore, the expansions of transposon-suppressive TF could have occurred in the past, beginning with the unicellular organisms, but only a minority of them, which adopted new functions, were retained.

For example, although ancient TF (with MGI < 0.1) were enriched significantly only in the latest phylostrata (Figure 6C), some of them were present in the Bilateria and the Euteleostomi (Figure 6B). They are also ZF-C2H2 genes but, instead of KRAB, they contain BTB/POZ or SET domains (Supplementary Table S4), which are also repressive and can suppress retroelements [57,58]. Thus, the expansions of transposon-suppressive TF with the adoption of a minority of them for other organism’s needs probably accompanied the evolution from the beginning of cellular life. Whether they were related to biological complexity is an open question. This is doubtful with regard to their latest expansion because birds have about 20-fold lesser KRAB genes than reptiles (Supplementary Table S3). Most likely, the abundance of KRAB genes depends on recent transposon expansions and relates to clades rather than to grades of complexity.

### 3.4. Salient Shift in Molecular Mechanisms after Euteleostomi

Both the regulatory gene families and the complexity-related gene properties showed a sharp decline after the Euteleostomi, which suggests that other evolutionary factors became important. The change of factors could be related to a change in the anatomical evolution. Nearly all recent phyla had already appeared at the Cambrian explosion [59], which suggests that after the main body plans were established, the evolution was constrained in its possibilities, becoming a variation on a theme.

However, the growth of complexity in the vertebrate line still continued, as can be seen from the homeothermy and the four-chambered heart, with complete separation into two pumps that were independently acquired by birds and mammals [60]. The increase in the encephalization quotient, and the appearance and extension of neocortex and its cognitive parts in mammals (especially in hominids), are the other indicators [15,48].

Genes that originated after the Euteleostomi showed a twofold higher rate of coding sequence change, which suggests a stronger involvement of protein sequence evolution (Figure 9). Thus, several lines of evidence indicate a salient shift in the molecular mechanisms of evolution after the Euteleostomi (when the basic vertebrate body plan was fixed with the bony skeleton). Notably, this evolutionary stage roughly corresponds to the ‘phylotypic’ developmental stage when the body plan was formed and the vertebrate embryos most closely resembled each other [61]. (More precisely, only the euteleostome embryos were studied and contrasted with the embryos of non-vertebrate chordates.) This correspondence indicates that this stage is special for both development and evolution.

Besides the changes in protein sequences, the possible mechanism of further growth of complexity is the changes in gene expression. They were involved in the growth of the human brain [48,62,63]. It was supposed that at small phylogenetic distances (i.e., at last evolutionary stages), the changes in gene expression can be more important than the changes in protein-coding sequences [18,19]. In the human–primate and human–mouse comparisons, the systemic expression changes are most prominent for the genes involved in translation [64]. Translational regulation is associated with cell specialization because it allows a faster fine-tuning of protein repertoire as compared with transcriptional regulation [65]. The growth of cell specialization should increase the number of cell types. In agreement with the increasing cell specialization at the later stages of evolution, there is an increase in the tissue-specificity of younger genes (Supplementary Figure S7). Notably, the highest gene complexity was previously found in the genes with an intermediate expression breadth [66], which roughly corresponds to the intermediate phylostrata (Metazoa-Euteleostomi), where many complexity-related gene properties showed their maximum values. These genes probably served as a connection between the earlier- and later-appearing genes in the work of cell systems.

### 3.5. Summary of the Main Points

(1) Methodologically, we introduced the distinction between the ‘deep’ and ‘shallow’ phylostratigraphy, compared these approaches, and discussed them in the context of the classic ‘lumping vs. splitting’ problem (thereby extending it to the molecular field). This helps explain the controversial gene datings that were published previously.

(2) Surprisingly, although transcription factors (TF) originated in the Prokaryota while chromatin appeared only in the Eukaryota, the expansion of epigenetic factors predated the expansion of TF. Protein modifiers and epigenetic factors expanded at the unicellular evolutionary stage, whereas TF and signaling receptors expanded in the multicellular organisms. Because cancer is an atavistic shift to unicellularity, these observations suggest that protein modifiers and epigenetic factors can be (at least) as important for oncogenesis as TF and signaling receptors.

(3) The expansion of nervous system-related genes could create a misleading notion that the growth of complexity in the latest phylostrata was owing to these genes. However, this later expansion was due to olfactory receptors, which reflected only the elaboration of olfaction. Without olfactory receptors, the nervous system genes expanded only up to the Euteleostomi (bony vertebrates).

(4) Several lines of evidence suggest a salient shift in the evolution of complexity after the Euteleostomi. The expansions of regulatory gene families as well as the disease genes (indicating vulnerabilities in a complex organization and strongly enriched in regulatory genes) sharply declined after the Euteleostomi, with one paradoxical exception (explained below). The complexity-related gene properties (protein size, number of alternative splicing mRNA, length of untranslated mRNA, number of biological processes per gene, number of disordered regions in a protein, and density of TF–TF interactions) rose in multicellular organisms and declined after the Euteleostomi or earlier. At the same time, the speed of protein sequence evolution sharply increased.

(5) The above-mentioned exception is a unique expansion of TF families of deeply unicellular origin in the placentals, whereas the earlier expansion of TF was of purely multicellular origin. This inversion of TF origins and expansions creates a paradox, which can be explained as follows. It is likely that expansions of deeply unicellular TF occurred in the past, beginning from early times, but now we can see only their traces. They were situational expansions that occurred for the purpose of coping with invading transposons (‘nuclear immunity’) and deteriorated after the diminishing of the activity of cognate transposons. Only a minor part of these TF were adopted for other organism’s needs and retained (we showed the remnants of earlier expansions). Therefore, the recent expansion of deeply unicellular TF was related to clades rather than to grades of complexity. Thus, the expansions of olfactory and ‘nuclear immunity’ genes seem to be two possible pitfalls in the analysis of the impact of the gene repertoire on the growth of complexity.

## 4. Methods

### 4.1. Phylostratigraphy

Phylostratigraphy (gene dating), which is based on gene orthology, was proposed in [33]. Orthologs are genes from the different genomes that originated from a common ancestor gene by organism speciation, while paralogs originated within the same genome by gene duplication. We propose discerning the shallow phylostratigraphy (splitting), which uses strict orthology and reveals more recent gene dating, and the deep phylostratigraphy (lumping) that uses relaxed orthology and reveals an earlier origin of co-orthologs (in-paralogs). The relaxed orthology is presented by orthologous gene groups, which include in-paralogs arising by means of within-genome duplication after the split of phylogenetic lineages [67]. Notably, we used an orthology that was obtained with the exact Smith–Waterman algorithm, which guaranteed optimum alignment [68], and synteny (for later phylostrata). In addition, we did not study single genes, but rather functional gene groups where stochastic noise was reduced through the aggregation of many genes.

For the shallow phylostratigraphy (splitting), we combined the two sources: NCBI orthologs and the OMA database [69,70]. In total, there were 17 phylostrata, which are listed in the figure legends. The NCBI 1:1 orthology is based both on sequence similarity and synteny; hence, it was preferred. However, synteny was not preserved for a long time; therefore, NCBI provides orthology only for the later phylostrata. We used NCBI orthologs up to Mammalia (12–17 phylostrata). A human gene was assumed to appear at a phylostratum that contained the earliest branching from the human phyletic line with an ortholog to this human gene (e.g., if the earliest-branching species with an ortholog to human was the opossum, the gene was ascribed to Theria). Further in the past (1–11 phylostrata), we used the OMA orthology groups. The OMA orthologs were calculated using the best reciprocal hits (BRH) of all-against-all genes for pairwise genome comparisons made with the exact Smith–Waterman algorithm. An OMA group contains genes from different species that are strict orthologs to each other. We selected all OMA groups that contained a human gene. Similarly, with the NCBI case, the human gene was assumed to have appeared at a phylostratum that contained, in the OMA group, a gene from a species with the earliest branching from human phyletic line. The shallow phylostratum is provided in Supplementary Lists S1.

For the deep phylostratigraphy (lumping), we used the orthologous gene groups (OGG) from OrthoDB [71]. This database combines the accurate method of orthology determination (based on the exact Smith–Waterman algorithm) with the broadest species coverage (1271 eukaryotes). The broad coverage is important for phylostratigraphy because a gene can be lost in some lineages and the insufficient species coverage would shift the dating to more recent times. The OrthoDB OGGs are obtained by a multi-step procedure based on the BRH of all-against-all genes. After the determination of BRH, the matches within each genome that are more similar than the BRH between the genomes are identified (they represent gene duplications that occurred after this branching point, i.e., co-orthologs/in-paralogs). The third and final step involves triangulating and clustering all BRH and in-paralogs into the groups of orthologous genes (OGG). Such clusters represent all descendants of a presumably single gene of the last common ancestor (LCA) of all species considered. In other words, these genes are the relatives but not necessarily the direct descendants of LCA by speciation (as the strict orthologs).

Taking into account that the main contradiction of multicellularity is between the cellular and organismal levels [25], we constructed the multicellularity gene index (MGI), which is defined as the proportion of multicellular species in the OGG containing a human gene. In other words, MGI shows how typical a gene is for multicellular organisms as compared to unicellular organisms. In addition, MGI can be considered as a measure of the confidence of multicellularity assignment. The determination of orthology may be controversial due to statistical errors (because of spurious sequence matches or insufficient species coverage). However, a low MGI (i.e., a high proportion of unicellular species in the OGG) indicates that they are not spurious matches and the gene indeed appeared at the unicellular evolutionary stage. The presentation of species in the OGGs was balanced to remove a bias in favor of taxa with many sequenced genomes. This was achieved by restricting the number of top taxonomic levels in the classification of species presented in an OGG. We chose the eighth upper level of the NCBI taxonomy (e.g., all Chordata were counted in an OGG as only one count). Therefore, there was no bias in favor of mammals, insects, teleosts, and other clades with plenty of sequenced genomes. This procedure did not qualitatively affect the picture. In particular, it did not change the results when contrasting genes with MGI < 1 (deeply unicellular origin) and MGI = 1 (purely multicellular origin).

To embrace both unicellular and multicellular organisms, we chose the OGGs rooted at the Eukaryota. The multicellular plants and fungi were excluded to avoid complicating the picture with non-animal multicellularity, which can be principally different (e.g., the presence of cell envelopes). Therefore, this was a strictly animal MGI, which was more suitable for this work. The MGI (and corresponding OGG) is provided in Supplementary List S2.

### 4.2. Gene Repertoire Analysis

In this approach, we analyzed the proportion of genes belonging to the various gene groups (genes united by a certain characteristic, see below) in the sets of genes that originated at different phylostrata. This proportion was compared with the proportion of genes belonging to this group in the whole genome. Thus, the whole-genome proportion was used as a baseline for a given gene group. In the integrated analyses, all baselines of the compared gene groups were set to zero. The analyses were conducted with the Statgraphics Centurion XVI software package.

The gene groups were as follows: Gene Ontology ‘protein modification process’ GO:0036211, ‘kinase activity’ GO:0016301, ‘signaling receptor activity’ GO:0038023, ‘nervous system process’ GO:0050877, ‘olfactory receptor activity’ GO:0004984, ‘regulation of multicellular organismal process’ GO:0051239, ‘central nervous system development’ GO:0007417, as well as the attribution to any GO biological process containing the word ‘brain’ or ‘regulation’, and to any GO cell component containing the word ‘neuron’. GO categories were taken from [72]. For each GO category, we collected all its subcategories using GO acyclic directed graphs, and a gene was regarded as belonging to a given category if it was mapped to any of its subcategories.

The main set of transcription factors (TF) and the set of TF cofactors were taken from [73]. In addition, two other TF sources were used: from [50] and ‘DNA-binding transcription factor activity’ GO:0003700. The epigenetic factors were obtained from the EpiFactors database [74]. The chaperones were derived from [37]. The bivalent genes (Fantom-confirmed) were obtained from [38]. The different cancer-related signatures were obtained from TSGene, NCG, COSMIC, and BioSystems [69,75,76,77]. The pluripotency signatures were obtained from PluriNet [78] and the set of genes upregulated in human embryonic stem cells (ESC) in at least three independent studies [79]. These pluripotency signatures were checked in the single-cell ESC transcriptomes with the control for cell cycle activity [26]. The age- and disease-related genes were obtained from HAGR and DisGeNET [80,81]. The ohnologs were acquired from the OHNOLOGS database [42].

### 4.3. Other Analyses

The number of alternative splicing mRNA variants, protein length (maximal for a gene), and UTR length were determined from mRNA sequences using the non-redundant RefSeq database [69]. The attribution of InterPro domains and protein disordered regions to the genes was taken from Ensembl [82]. The numbers of olfactory receptors and visual-perception genes in different mammals were determined from GO attribution in Ensembl. The protein interactions were derived from STRING [83]. Gene expression (mRNA levels) in normal tissues were obtained from Human Protein Atlas [84].

The enrichment of pathways, GO categories and InterPro domains (shown in Supplementary Tables S1–S5) was estimated as described previously [25,64]. The pathways were taken from BioSystems [69]. Redundancy of this resource, which is a most complete compendium of pathways from different sources, was removed by uniting entries with identical gene sets. The hypergeometric distribution of probability (implemented in the R environment) was used for determination of the statistical significance of the ratio of observed-to-expected numbers of genes belonging to a pathway or a GO category (or containing an InterPro domain) in a tested gene sample. The expected number was calculated based on the number of pathway/category/domain genes in total gene dataset (assuming random gene distribution across pathways/categories/domains). After the determination of enriched pathways/categories/domains, the statistical significance of enrichment was corrected for multiple tests according to [85].

The protein evolutionary rates (*dN*) were determined as previously [3]. The coding sequences were taken from RefSeq for human, macaque, mouse, and cow (the latter was chosen as an outgroup because it is a best-studied genome in proximity to the primate/rodent clade). They were aligned separately in the two triads of orthologs (human–macaque–mouse and human–mouse–cow) using the PRANK program with a phylogenetic guide tree [86]. The forced phylogeny option (+F) was used. The alignments were conducted at the codon level because PRANK performs better in this way [86]. The rates of nonsynonymous (*dN*) nucleotide substitutions were determined using the probabilistic model of codon evolution implemented in ‘codeml’ from the PAML package [87]. We determined *dN* for alignments of ancestral sequences with human sequences and for direct human–cow comparison. The ancestral sequences were reconstructed on the grounds of hidden Markov models using PRANK (‘-showanc’ option). They were the human–macaque ancestor (with the mouse as a reference outgroup) and the human–mouse ancestor (with the cow as an outgroup).

## 5. Conclusions

We concluded that the molecular mechanisms of complexity growth were changing with time, and after the basic vertebrate body plan was fixed with bony skeleton (in the Euteleostomi), there was a salient shift in these mechanisms within the phyletic lineage leading to humans. The first growth wave involved the expansion of protein modifiers (including epigenetic factors) that represented regulation at the level of gene products. They showed a plateau at the Eukaryota-Metazoa (unicellular sponges) and a depression after the Chordata. The second wave involved signaling receptors (representing intercellular communication), closely followed by a wave of transcription factors (probably representing diversification of cell types). These genes showed significant enrichment at the Bilateria and decline after the Euteleostomi. The wave of the nervous system genes generally coincided with the growth of other regulatory genes. This was probably because the nervous system should integrate all the other complexity waves at the organismal level. After the Euteleostomi, we revealed no significant expansions in gene groups that were related to general complexity (albeit there was an elaboration of olfaction and ‘nuclear immunity’). The overall picture of the main changes in the regulatory gene repertoire is shown in Figure 4.

The post-Euteleostomi complexity growth probably proceeded mostly via changes in protein sequences (the speed of which sharply increased) and in gene expression, with small-scale changes in the gene repertoire. The changes in gene expression could have been affected by a multitude of means, including changes in cis-regulatory elements, non-coding RNA, the structure of chromatin, and complex interactions of TF between themselves and with TF cofactors and epigenetic factors. The changes in TF complexes could have affected both transcription initiation and promoter-proximal pausing [88]. The invasion and propagation of transposable elements could also have changed the gene expression patterns [89,90]. Furthermore, there could have arisen intricate combinations of variation in terms of non-coding DNA, chromatin structure, TF, and epigenetic factors (with a growing space of splicing variants), followed by elaborate (post-) translational regulation.

Beginning from a certain threshold number of the basic genetic elements (protein-coding genes), the *combinatorial space* of protein products of these elements may have become large enough such that the further growth of organismal complexity could proceed without significant expansion in the genetic elements’ base. Natural selection explores this combinatorial space via changes in gene expression and protein interactions. Finally, the expansion of regulation to the higher organization level (from regulatory genes to regulatory cells) seemed to be involved, which was manifested in the increase in the number of neurons (approximated by encephalization quotient).

All these possibilities cannot so far be traced, in the evolutionary path leading to humans, in a consistent and quantitative way that is similar to that of protein-coding sequences. Therefore, we should include a caveat that our approach embraces only a part of the possible mechanisms of complexity growth. In the future, other aspects can be added to this picture.

## Data Availability

The data underlying this article are available in the article and its online Supplementary Materials.

## Supplementary Materials

The following are available online at https://www.mdpi.com/article/10.3390/ijms222111640/s1.
