# Supplementary material for: Growth of Biological Complexity from Prokaryotes to Hominids Reflected in the Human Genome
Source: Int J Mol Sci. 2021 Oct 28;22(21):11640. doi: 10.3390/ijms222111640 (PMC8583824; doi:10.3390/ijms222111640)
Supplement: Supplementary file 1 [file ijms-22-11640-s001.zip › vinogradov_SupFigs.pdf]

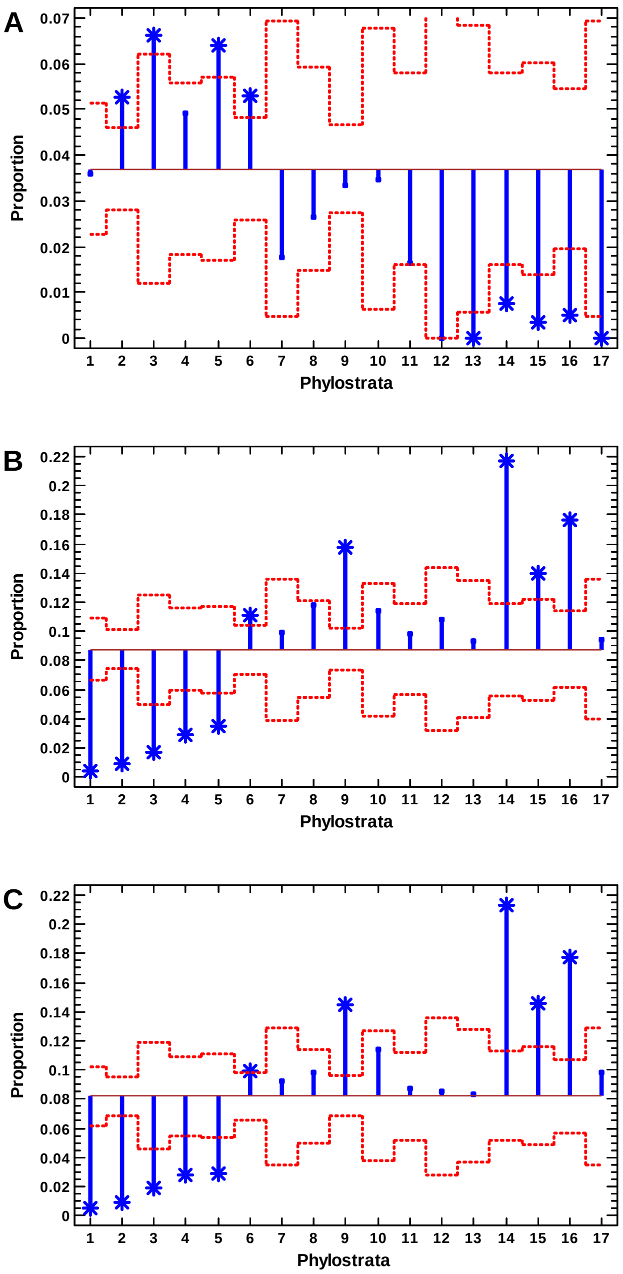

Sup. Figure S1. Gene proportion analysis of **protein kinases** (**A**), **TF from** Lambert et al. 2018 (**B**), and **TF from** 'DNA-binding transcription factor activity' GO:0003700 (**C**). Red dotted lines show significance level (p<0.05), asterisks show significant enrichment (if above baseline) or underrepresentation (below baseline). (1- cellular organisms, 2 - Eukaryota, 3 - Opisthokonta, 4 - Metazoa, 5 - Eumetazoa, 6 - Bilateria, 7 - Chordata, 8 - Vertebrata, 9 - Euteleostomi, 10 - Tetrapoda, 11 - Amniota, 12 - Mammalia, 13 - Theria, 14 - Eutheria, 15 - Boreoeutheria, 16 - Primates, 17 - Hominidae.)

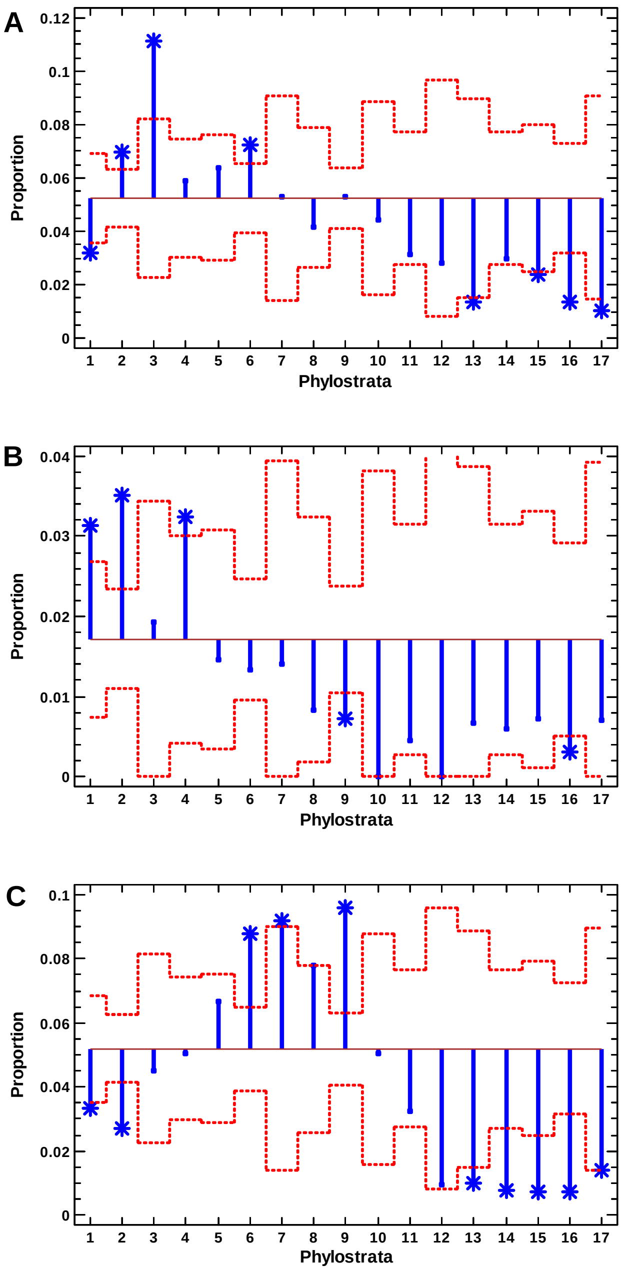

Sup. Figure S2. Gene proportion analysis of **TF cofactors** (**A**), **chaperones** (**B**), and **bivalent genes** (**C**). Red dotted lines show significance level (p<0.05), asterisks show significant enrichment (if above baseline) or underrepresentation (below baseline). (1- cellular organisms, 2 - Eukaryota, 3 - Opisthokonta, 4 - Metazoa, 5 - Eumetazoa, 6 - Bilateria, 7 - Chordata, 8 - Vertebrata, 9 -Euteleostomi, 10 - Tetrapoda, 11 - Amniota, 12 - Mammalia, 13 -- Theria, 14 - Eutheria, 15 -Boreoeutheria, 16 - Primates, 17 - Hominidae.)

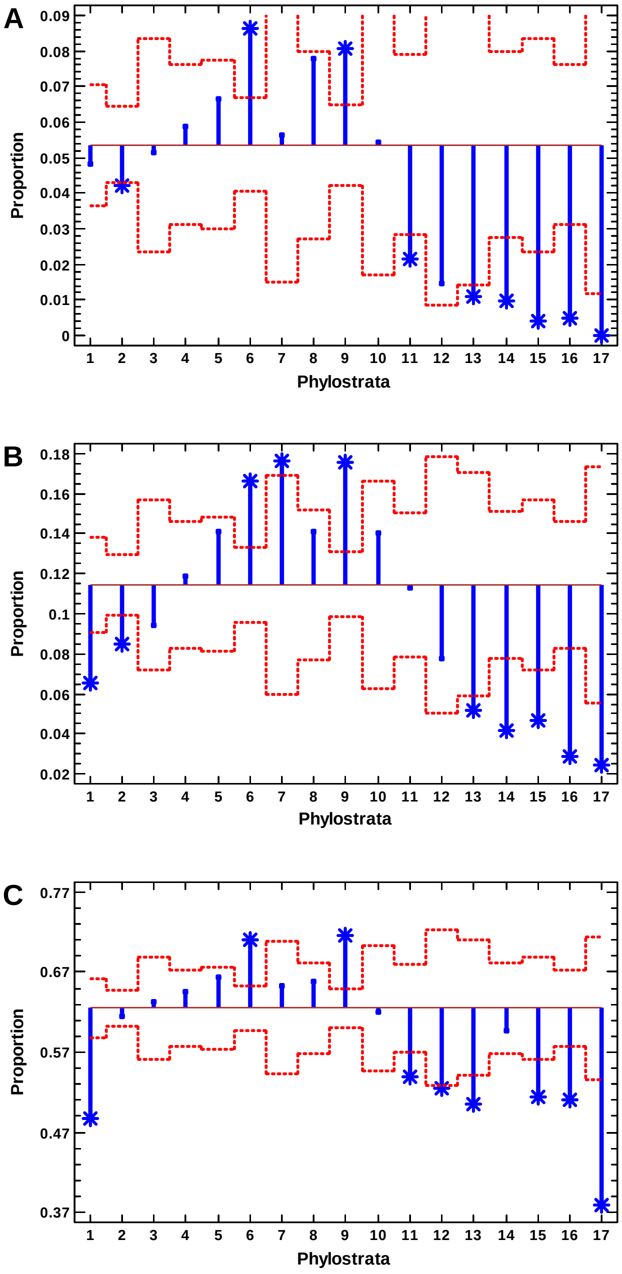

Sup. Figure S3. Gene proportion analysis of **CNS development** genes (**A**), **'regulation of multicellular organismal process'** genes (**B**), and any GO biological process containing a word **'regulation'** (**C**). Red dotted lines show significance level (*p*<0.05), asterisks show significant enrichment (if above baseline) or underrepresentation (below baseline). (1- cellular organisms, 2 - Eukaryota, 3 - Opisthokonta, 4 - Metazoa, 5 - Eumetazoa, 6 - Bilateria, 7 - Chordata, 8 - Vertebrata, 9 - Euteleostomi, 10 - Tetrapoda, 11 - Amniota, 12 - Mammalia, 13 - Theria, 14 - Eutheria, 15 - Boreoeutheria, 16 - Primates, 17 - Hominidae.)

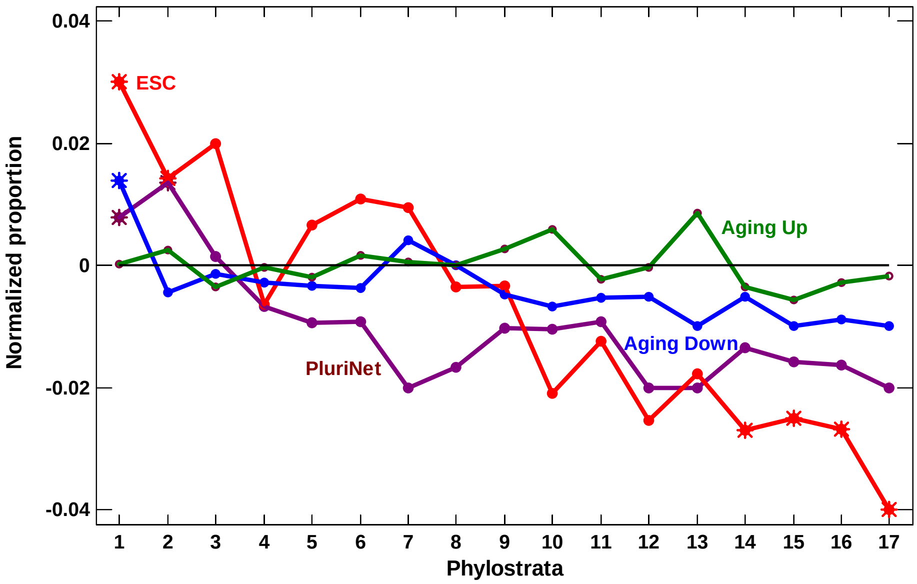

Sup. Figure S4. Phylostratic course of normalized gene proportions for pluripotency and aging signatures (baselines for all gene groups are set to zero). Asterisks show significant enrichment (if above baseline) or underrepresentation (below baseline). (1- cellular organisms, 2 - Eukaryota, 3 - Opisthokonta, 4 - Metazoa, 5 - Eumetazoa, 6 - Bilateria, 7 - Chordata, 8 - Vertebrata, 9 - Euteleostomi, 10 - Tetrapoda, 11 - Amniota, 12 - Mammalia, 13 - Theria, 14 - Eutheria, 15 - Boreoeutheria, 16 - Primates, 17 - Hominidae.)

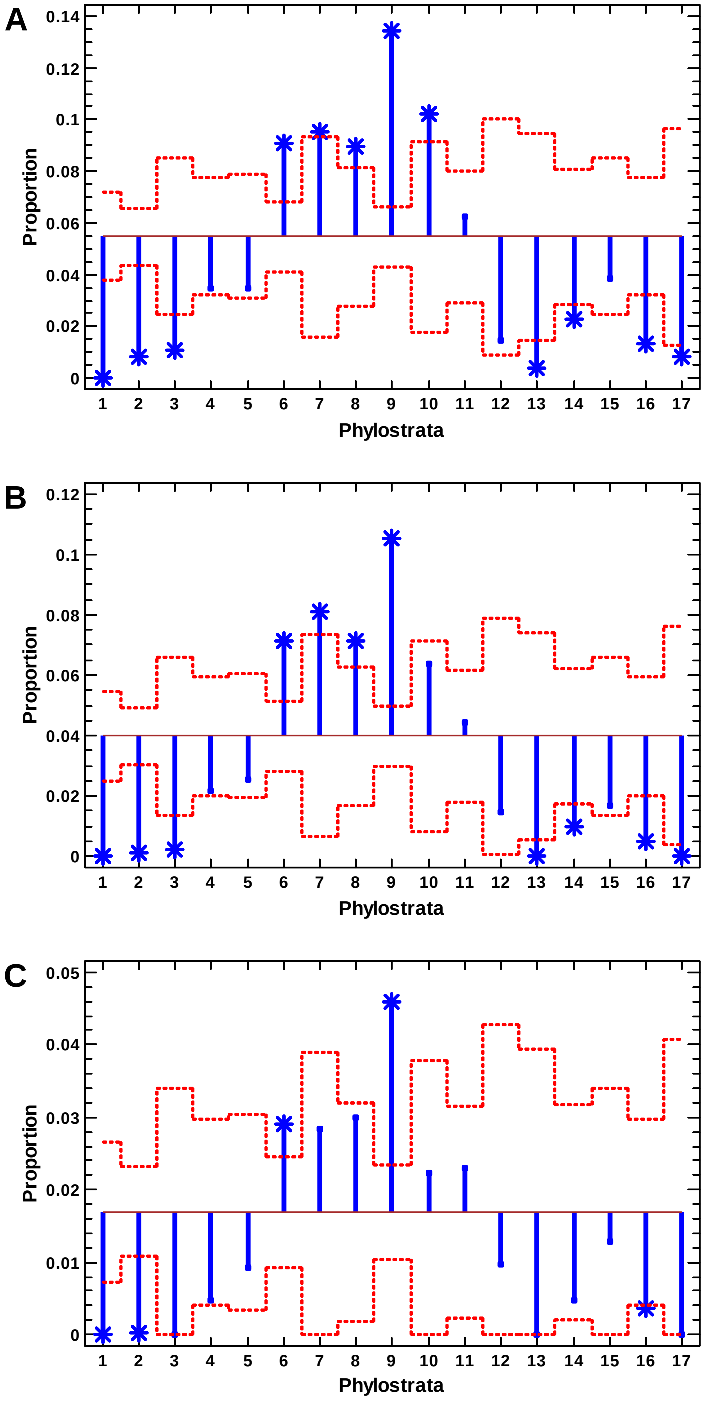

Sup. Figure S5. Gene proportion analysis of transcription factors (TF) with different multicellularity gene index (MGI). **A -- MGI>0.1**. **B -- MGI>0.5**. **C -- MGI=1**. In fact, this is presentation of data in Fig. 6B using different slices along the MGI axis and indicating significance level. Red dotted lines show significance level (*p*<0.05), asterisks show significant enrichment (if above baseline) or underrepresentation (below baseline). (1- cellular organisms, 2 - Eukaryota, 3 - Opisthokonta, 4 - Metazoa, 5 - Eumetazoa, 6 - Bilateria, 7 - Chordata, 8 - Vertebrata, 9 - Euteleostomi, 10 - Tetrapoda, 11 - Amniota, 12 - Mammalia, 13 - Theria, 14 - Eutheria, 15 - Boreoeutheria, 16 - Primates, 17 - Hominidae.)

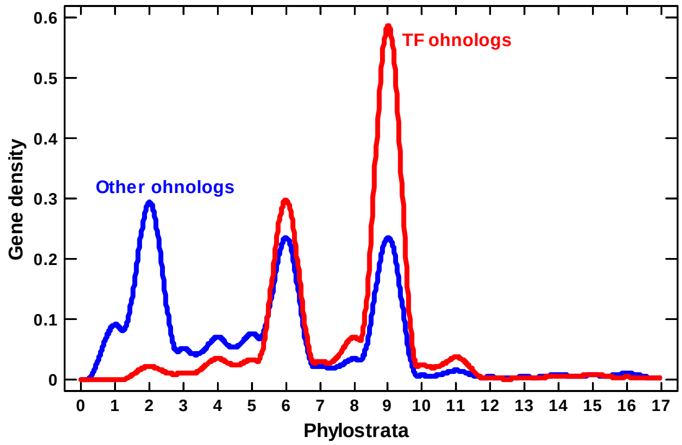

Sup. Figure S6. Gene density distributions of TF-ohnologs and other ohnologs across phylostrata. For difference at Euteleostomi, $p<10^{-54}$ (hypergeometric test). (1- cellular organisms, 2 - Eukaryota, 3 - Opisthokonta, 4 - Metazoa, 5 - Eumetazoa, 6 - Bilateria, 7 - Chordata, 8 - Vertebrata, 9 - Euteleostomi, 10 - Tetrapoda, 11 - Amniota, 12 - Mammalia, 13 - Theria, 14 - Eutheria, 15 - Boreoeutheria, 16 - Primates, 17 - Hominidae.)

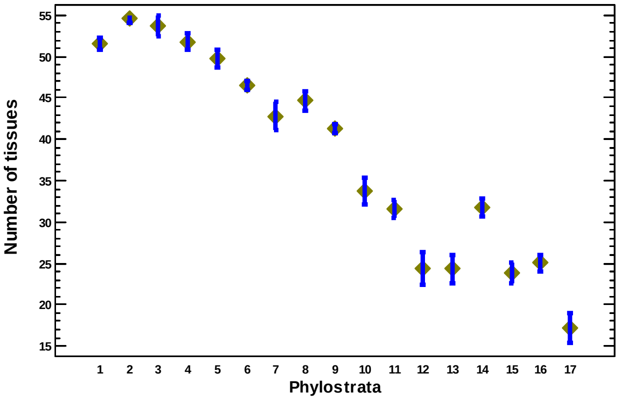

Sup. Figure S7. Phylostratic course of the number of tissues where a gene is expressed. Means with LSD intervals (p<0.05). (1- cellular organisms, 2 - Eukaryota, 3 - Opisthokonta, 4 - Metazoa, 5 - Eumetazoa, 6 - Bilateria, 7 - Chordata, 8 - Vertebrata, 9 - Euteleostomi, 10 - Tetrapoda, 11 - Amniota, 12 - Mammalia, 13 - Theria, 14 - Eutheria, 15 - Boreoeutheria, 16 - Primates, 17 - Hominidae.)
